# Plasma metabolomics for the preoperative diagnosis of parathyroid carcinoma

**DOI:** 10.1530/ERC-24-0192

**Published:** 2025-07-01

**Authors:** Jinheng Xiao, Ming Cui, Qingyuan Zheng, Sen Yang, Tianqi Chen, Ya Hu

**Affiliations:** ^1^ Department of General Surgery, State Key Laboratory of Complex Severe and Rare Disease, Peking Union Medical College Hospital, Chinese Academy of Medical Sciences & Peking Union Medical College, Beijing, China; ^2^ Medical Research Center, Peking Union Medical College Hospital, Chinese Academy of Medical Sciences & Peking Union Medical College, Beijing, China

**Keywords:** parathyroid carcinoma, parathyroid adenoma, metabolomics, tryptophan, 7-ketodeoxycholic acid

## Abstract

Parathyroid carcinoma (PC) is a rare endocrine malignancy with a poor prognosis. Preoperative diagnosis remains a major challenge in clinical practice because of limited diagnostic methods and non-specific clinical features. This study aimed to describe the distinct plasma metabolic profiles of PC and parathyroid adenoma (PA) patients and identify promising biomarkers for the preoperative differential diagnosis of PC. A total of 115 patients were enrolled in this retrospective study, including 70 patients (24 PC and 46 PA) in the discovery cohort and 45 patients (15 PC and 30 PA) in the validation cohort. Plasma samples were collected before operation. LC–MS/MS analysis was utilised on the discovery cohort to explore the metabolic profile and find out differentially abundant metabolites. Subsequently, potential diagnostic biomarkers were verified in an external validation cohort to find out novel biomarkers for the differential diagnosis of PC before surgery. Compared with the plasma samples of PA patients, a total of three upregulated and 42 downregulated metabolites were identified by MS/MS in the plasma samples of PC patients. The differentially abundant metabolites were significantly enriched in the arachidonic acid, tryptophan, and hormone metabolism pathways. Notably, 7-ketodeoxycholic acid (*P* = 0.002, AUC = 0.804) and tryptophan (*P* < 0.001, AUC = 0.838) were confirmed to show high accuracy in differential diagnosis in the validation cohort. Therefore, metabolomics analysis of parathyroid neoplasms revealed significant differences in metabolic profiles between PAs and PCs. The plasma levels of 7-ketodeoxycholic acid and tryptophan could serve as potential diagnostic biomarkers for PC.

## Introduction

Primary hyperparathyroidism (PHPT) is the third most common endocrine disorder in humans. Excessive secretion of parathyroid hormone (PTH) can cause hypercalcaemia, leading to a variety of complications, such as renal calculus, chronic kidney failure, severe osteoporosis, and even hypercalcaemic crisis. Most PHPTs are caused by parathyroid neoplasms. According to the 2022 WHO pathological classification of parathyroid neoplasms, parathyroid neoplasms are divided into parathyroid adenoma (PA), atypical parathyroid tumour (APT), and parathyroid carcinoma (PC) (Erickson *et al.* 2022).

PC is a rare endocrine malignancy that accounts for approximately 1% of sporadic PHPT (Cetani *et al.* 2019). Precise preoperative diagnosis is crucial for determining the extent of surgical resection. En bloc resection of the tumour, ipsilateral thyroid, and even central lymph nodes is suggested for PC, while local tumour resection is adequate for PA (Salcuni *et al.* 2018). A misdiagnosis as PC leads to excessive surgical extension, which may result in severe surgical complications; and a missed diagnosis of PC causes an inadequate extent of surgery, which may increase the risk of tumour residue, recurrence, or metastasis. Even if larger tumours or high serum levels of PTH and calcium indicate a risk of malignancy, the preoperative diagnosis of PC remains a major challenge in clinical practice (Betea *et al.* 2015). There are significant overlaps in clinical presentations and histopathological features between PAs and PCs, especially among PA patients after long-term delay of diagnosis (Talat & Schulte 2010). Unlike for thyroid neoplasms, preoperative fine-needle aspiration biopsy is not recommended for parathyroid neoplasms due to the possible risk of tumour cell dissemination, haemorrhage, and low diagnostic accuracy (Wilhelm *et al.* 2016). Therefore, characteristic features or biomarkers in plasma are needed to improve the preoperative diagnostic efficacy for PCs. In this context, numerous groundbreaking explorations have been reported previously. A 3rd/2nd generation plasma PTH ratio >1 could identify PCs earlier, with a sensitivity of 83.3% and a specificity of 100% (Cavalier *et al.* 2010). In addition, it was reported that the high level of the malignant hCG hyperglycosylated isoform might provide new evidence for the diagnosis of PCs (Rubin *et al.* 2008). Besides, the higher expression of long non-coding RNAs BC200 in serum from PC patients could be regarded as a useful biomarker for diagnosis (Morotti *et al.* 2022).

Metabolomics is an emerging technology that can help to identify biomarkers of malignancies and reveal driving factors of tumourigenesis, which has attracted great attention in recent years (Schmidt *et al.* 2021). Metabolomics has been applied to cancer screening or diagnosis of a variety of tumours, including lung cancer, pancreatic cancer, and colon cancer (Fan *et al.* 2009, Zhou *et al.* 2012, Manna *et al.* 2014). In 2016, Battini compared the metabolic profile of PHPT tissue samples with that of renal hyperparathyroidism by magnetic resonance spectroscopy, and a bicomponent orthogonal partial least square-discriminant analysis (OPLS-DA) model was constructed to distinguish single-gland parathyroid disease from multiglandular parathyroid disease (Battini *et al.* 2016). In another study, several environmental chemicals had been detected in parathyroid tumour tissues by gas chromatography-mass spectrometry/mass spectrometry (GC–MS/MS), which were involved in metabolic pathways in parathyroid tumour (Hu *et al.* 2021*a*). Recently, metabolic profiling of parathyroid organoids was explored, and the feasibility of using parathyroid organoids for metabolic research was confirmed (Sekhar *et al.* 2023). However, the plasma metabolic characteristics of PC patients have not yet been explored.

In this study, we conducted liquid chromatography and mass spectrometry (LC–MS/MS) analysis to identify distinct metabolic profiles in the plasma of PC and PA patients in the discovery cohort. In the validation cohort, the diagnostic values of several candidate biomarkers were further validated.

## Materials and methods

### Patients and samples

A total of 115 patients were included in this retrospective study, including 39 PC patients and 76 PA patients. All patients accepted surgery in Peking Union Medical College Hospital (PUMCH), and pathological diagnosis was determined by two endocrine pathologists according to the 2022 WHO Classification (Erickson *et al.* 2022). The histological definition of PC meets one of the following criteria: i) angioinvasion, ii) lymphatic metastasis, iii) intraneural invasion; iv) local invasion into adjacent anatomic structures; or v) distant metastasis (Erickson *et al.* 2022). Other selection criteria included: i) the age >18 and <75; ii) the patient did not suffer from kidney failure or liver failure; iii) the patient did not take medicines that affect the metabolism of substances in plasma, such as antibiotics or corticosteroids, within 1 month before blood draw; iv) no concomitant malignancies. Blood samples used in this study were collected from all patients before surgery. Plasma samples were immediately isolated from blood samples via centrifugation and stored at −80°C until use in experiments. The study was approved by the institutional ethics committee, and written informed consent was obtained from all 115 patients.

### Metabolite extraction and LC–MS/MS analysis

A total of 100 μL of plasma and 300 μL of extraction solution (methanol, containing an isotopically labelled internal standard mixture) were mixed and vortexed for 30 s. The mixture was then sonicated for 10 min in an ice-water bath and incubated for 1 h at −40°C to precipitate proteins. Then, the sample was centrifuged at 1.38 × 10^4^ *g* for 15 min at 4°C. The resulting supernatant was collected for further analysis.

LC–MS/MS analyses were performed using a UHPLC system (Vanquish, Thermo Fisher Scientific, USA) with a UPLC HSS T3 column (2.1 × 100 mm, 1.8 μm) coupled to an Orbitrap Exploris 120 mass spectrometer (Orbitrap MS, Thermo). Phase A consisted of 5 mmol/L ammonium acetate and 5 mmol/L acetic acid in water, and phase B consisted of acetonitrile. The rate of gradient elution was as follows: 0–0.7 min, 1% B, flow 0.35 mL/min; 0.7–9.5 min, 1–99% B, flow 0.35 mL/min; 9.5–11.8 min, 99% B, flow 0.35–0.5 mL/min; 11.8–12 min, 99%–1% B, flow 0.5 mL/min; 12–14.6 min, 1% B, flow 0.5 mL/min; 14.6–14.8 min, 1% B, flow 0.5–0.35 mL/min; 14.8–15 min, 1% B, flow 0.35 mL/min. The autosampler temperature was set to 4°C, and the injection volume was 2 μL. An Orbitrap Exploris 120 mass spectrometer was used for its ability to acquire MS/MS spectra in information-dependent acquisition (IDA) mode via acquisition software (Xcalibur, Thermo). In this mode, the acquisition software continuously evaluates the full-scan MS spectrum. The ESI source conditions were set as follows: sheath gas flow rate, 50Arb; aux gas flow rate, 15Arb; capillary temperature, 320°C; full MS resolution, 60,000; MS/MS resolution, 15,000; collision energy, 10/30/60 in NCE mode; and spray voltage, 3.8 kV (positive) or −3.4 kV (negative).

The raw data were converted to the mzXML format using ProteoWizard and processed with an in-house programme, which was developed using R and based on XCMS, for peak detection, extraction, alignment, and integration. Then, an in-house MS2 database (BiotreeDB, V2.1, Shanghai, China) was used for metabolite annotation. The cut-off for annotation was set at 0.3.

### Immunohistochemical (IHC) staining and CDC73 sequencing

The parafibromin staining and *CDC73* sequencing results for 60 parathyroid tumour tissues, including 23 PCs and 37 PAs, were collected from previous studies (Hu *et al.* 2020, 2021*b*, 2022). Other newly enrolled tumour specimens were routinely stained for parafibromin using methods described in previous studies (Hu *et al.* 2022). After deparaffinisation and dehydration, tissue sections from FFPE tumour samples were incubated in a 3% H_2_O_2_ solution. The antigen was retrieved with ethylenediaminetetraacetic acid, and 3% bovine serum albumin was used to block non-specific antibody binding sites. Primary antibodies against parafibromin (ab223840, Abcam, UK) were incubated with the slides overnight at 4°C. Secondary antibodies and diaminobenzidine were used for staining. The total loss of parafibromin staining was recognised as negative staining, with vascular endothelial cells and stromal cells serving as internal positive controls. *CDC73* mutations were defined as somatic or germline mutations according to next-generation sequencing. Parafibromin loss or *CDC73* mutations were identified as *CDC73* abnormalities. For patients whose parafibromin the IHC and *CDC73* sequencing results were inconsistent, IHC result was used for further analysis.

### Statistical analysis

Continuous variables and categorical variables are presented as the mean value ± standard deviation (SD), and case numbers or corresponding percentages, respectively. Differences among patient groups were tested by χ^2^ tests for categorical variables and *t*-tests for continuous variables. Correlations among metabolites were analysed by Pearson correlation. The relationships between differentially expressed metabolites and *CDC73* abnormalities were assessed by *t*-tests. Cox regression analysis was used to evaluate the influence of metabolites on disease-free survival (DFS) and overall survival (OS) outcomes. Metabolites with *P* < 0.05 in the univariate analysis were subjected to multivariate analysis. The screening criteria for differentially abundant metabolites were a *P* value < 0.05 and a variable importance in the projection (VIP) > 1. Further screening for promising biomarkers was mainly based on receiver operating characteristic (ROC) analysis, with an area under the curve (AUC) > 0.65. All statistical and ROC analyses were performed with SPSS Statistics for Windows version 16 (SPSS Inc., USA). Based on the GPower (3.1.9.2) software, we calculated the power of the study to be 0.96.

## Results

### The characteristic features of patients

A total of 46 PA patients and 24 PC patients were enrolled in the discovery cohort of this retrospective study. Significant differences in age (*P* = 0.040), serum calcium (*P* = 0.001), serum phosphorus (*P* = 0.001), and *CDC73* abnormalities (*P* < 0.001) were found between PC and PA patients. However, there was no difference in the sex ratio (*P* = 0.292), serum ALP (*P* = 0.251), and creatinine level (*P* = 0.499) (Table 1). Among 24 PC patients, 14 patients had experienced local recurrence or distant metastasis when they visited our group. Significant differences in serum calcium level (*P* = 0.020), serum PTH level (*P* = 0.039), total 25-hydroxyvitamin D level (*P* = 0.008), and tumour diameter (*P* = 0.013) were found between patients with initial tumours and patients with recurrent tumours. Compared to patients with initial tumours, patients with recurrent tumours showed lower serum PTH level, lower serum calcium level, and smaller tumour diameter. A total of seven patients experienced local recurrence, seven patients experienced distant metastasis after initial surgery, and one patient was found to have lung metastasis at the first hospital visit. Two patients did not achieve complete biochemical remission after initial surgery. Three PC patients (3/24) died from hypercalcaemia, with the median follow-up time of 69.70 months (range 39.9–177.5 months).

**Table 1:** Clinical characteristics of the patients included in the discovery cohort (*n* = 70 unless otherwise specified).

Characteristic features	PC		PA	*P* values
Case number (*n* = 70)	24		46	-
Male: female	8:16		10:36	0.292
Average age at diagnosis (years)	45.27 ± 13.85		55.15 ± 12.50	0.040*
BMI (kg/m^2^)	23.60 ± 3.01		23.58 ± 3.13	0.973
Smoking (Y:N)	5:19		7:39	0.554
Alcohol (Y:N)	1:23		0:46	0.163
Serum calcium level (mmol/L)	3.30 ± 0.36^†^	0.020*^,^^§^	2.81 ± 0.32	<0.001*
	2.98 ± 0.27^‡^			0.072
Serum PTH (pg/mL)	1,208.94 ± 616.24^†^	0.039*^,^^§^	548.07 ± 644.56	0.005*
	572.82 ± 755.36^‡^			0.904
Serum phosphorus level (mmol/L) (*n* = 68)	0.64 ± 0.18		0.80 ± 0.17	0.001*
Serum ALP level (U/L) (*n* = 67)	492.14 ± 727.00		312.74 ± 515.31	0.251
Creatinine (μmol/L)	78.54 ± 39.14		72.48 ± 33.32	0.499
Total 25-hydroxyvitamin D (ng/mL) (*n* = 63)	9.91 ± 6.21^†^	0.008*^,^^§^	13.64 ± 4.89	0.048*
	16.93 ± 5.45^‡^			0.041*
Tumour diameter (cm) (*n* = 69)	3.60 ± 1.61^†^	0.013*^,^^§^	2.88 ± 1.41	0.157
	1.97 ± 1.26^‡^			0.041*
*CDC73* abnormality (P:N) (*n* = 65)	15:8		2:40	<0.001*
*CDC73* mutation (P:N) (*n* = 50)	11:5		1:33	<0.001*
Parafibromin staining (P:N) (*n* = 44)	8:15		20:1	<0.001*
DFS (months) (*n* = 21)	38.80 (13.3–91.4)		-	-
OS (months) (*n* = 24)	69.70 (39.9–177.5)		-	-

PC, parathyroid carcinoma; PA, parathyroid adenoma; BMI, body mass index; PTH, serum parathyroid hormone level; ALP, serum alkaline phosphatase level; P, positive; N, negative; Y, yes; N, no; DFS, disease-free survival; OS, overall survival.

* *P* < 0.050.

^†^ Initial PC tumour.

^‡^ Recurrent PC tumour.

^§^ Significantly different between initial and recurrent PC tumours.

### The metabolic landscape of plasma from patients with parathyroid neoplasms

**Figure 1:**
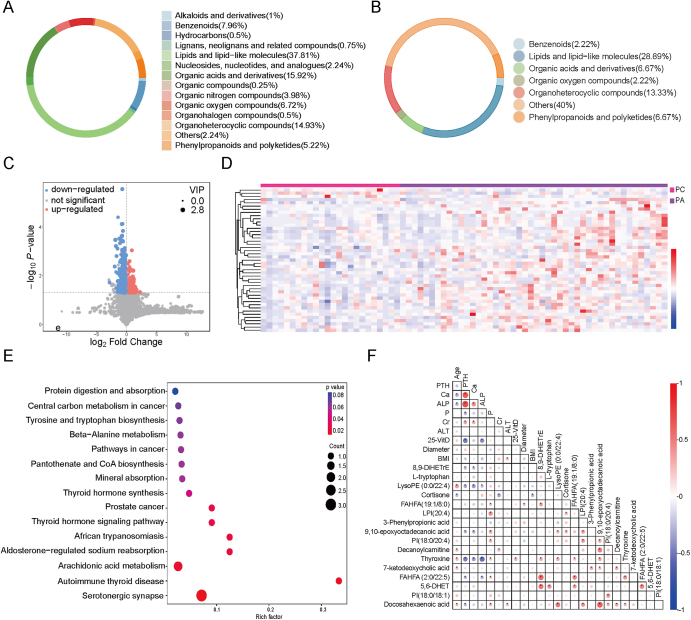
The metabolite profiles in plasma of patients with parathyroid neoplasm. (A) Circle plots showing the classification of total metabolites in plasma of patients with parathyroid neoplasm. (B) The classification of differential metabolites in plasma between patients with PC and PA. (C and D) Compared with the metabolites of the PA group, significantly upregulated and downregulated metabolites in the plasma of the PC group were shown in a volcano plot and a clustered heatmap plot. (E) KEGG enrichment bubble plot showing the differentially abundant metabolites enriched in several significant pathways. (F) Pearson correlations between clinical features and differentially abundant metabolites in plasma.

A total of 402 metabolites were identified in PC and PA patients based on the Human Metabolome Database (HMDB) and Kyoto Encyclopedia of Genes and Genomes (KEGG) compound databases. The top three types of metabolites were lipids and lipid-like molecules, organic acids and derivatives, and organoheterocyclic compounds, accounting for 37.81, 15.92, and 14.93% of metabolites, respectively (Fig. 1A). Based on the screening criteria, *P* value <0.05 and variable importance in the projection (VIP) > 1, a total of 45 differentially abundant metabolites were identified by MS/MS. Compared with the PA group, three metabolites were upregulated, and 42 metabolites were downregulated in the plasma of the PC group (Fig. 1B, C, D). These differentially abundant metabolites were enriched in 28 metabolic or regulatory pathways, and the top significant pathways involved endocrine hormone metabolism, thyroid hormone and cortisol metabolism, and substance metabolism, arachidonic acid, and tryptophan metabolism (Fig. 1E).

In the PC cohort, the metabolic profiles of patients with recurrent tumours were comparable to those of patients with initial tumours. A total of three metabolites showed significant differences, namely (R)-3-hydroxy-tetradecanoic acid (*P* = 0.041), 5Z-dodecenoic acid (*P* = 0.008), and isokobusone (*P* = 0.011). A total of 16 differentially abundant metabolites were associated with *CDC73* abnormalities (*P* < 0.05). Among them, lipids and lipid-like molecules, phenylpropanoids and polyketides, and organoheterocyclic compounds accounted for 31.25, 18.75, and 18.75%, respectively. The significant pathway (*P* < 0.05) was the arachidonic acid metabolism pathway, involving 5,6-DHET.

### Detection of potential biomarkers for distinguishing parathyroid carcinoma

In order to search for potential metabolic biomarkers for the discrimination of PC, the ROC curve was used to assess the diagnostic performance of the differentially abundant metabolites. Notably, 16 downregulated endogenous metabolites showed significant potential in differential diagnosis, including 8,9-DiHETrE (AUC = 0.762), L-tryptophan (AUC = 0.709), LysoPE (0:0/22:4) (AUC = 0.706), cortisone (AUC = 0.698), FAHFA (19:1/8:0) (AUC = 0.694), LPI (20:4) (AUC = 0.688), 3-phenylpropionic acid (AUC = 0.688), 9,10-epoxyoctadecanoic acid (AUC = 0.678), PI (18:0/20:4) (AUC = 0.677), decanoylcarnitine (AUC = 0.673), thyroxine (AUC = 0.672), 7-ketodeoxycholic acid (AUC = 0.671), FAHFA (2:0/22:5) (AUC = 0.668), 5,6-DHET (AUC = 0.667), PI (18:0/18:1) (AUC = 0.657), and docosahexaenoic acid (AUC = 0.656) (Table 2).

**Table 2:** Clinical characteristics of the patients included in the validation cohort (*n* = 45 unless otherwise specified).

Characteristic features	PC	PA	*P* values
Case number	15	30	-
Male: female	8:7	10:20	0.197
Average age at diagnosis (years)	49.27 ± 12.51	53.07 ± 14.84	0.399
BMI (kg/m^2^)	24.29 ± 2.86	25.62 ± 4.84	0.335
Smoking (Y:N)	6:9	5:25	0.086
Alcohol (Y:N)	4:11	3:27	0.146
Serum calcium level (mmol/L)	3.16 ± 0.40	2.74 ± 0.23	<0.001*
Serum PTH (pg/mL)	1,258.95 ± 1,036.79	305.94 ± 458.95	0.003*
Serum phosphorus level (mmol/L)	0.61 ± 0.21	0.89 ± 0.18	<0.001*
Serum ALP level (U/L)	459.13 ± 470.41	141.93 ± 135.50	0.022*
Creatinine (μmol/L)	75.13 ± 34.09	63.73 ± 17.59	0.240
Total 25-hydroxyvitamin D (ng/mL)	11.28 ± 4.07	17.28 ± 8.51	0.013*
Tumour diameter (cm)	2.46 ± 1.08	2.17 ± 0.86	0.333

PC, parathyroid carcinoma; PA, parathyroid adenoma; BMI, body mass index; PTH, serum parathyroid hormone level; ALP, serum alkaline phosphatase level; P, positive; N, negative; Y, yes; N, no.

* *P* < 0.050.

### Validation of plasma biomarkers in distinguishing parathyroid carcinoma

**Figure 2:**
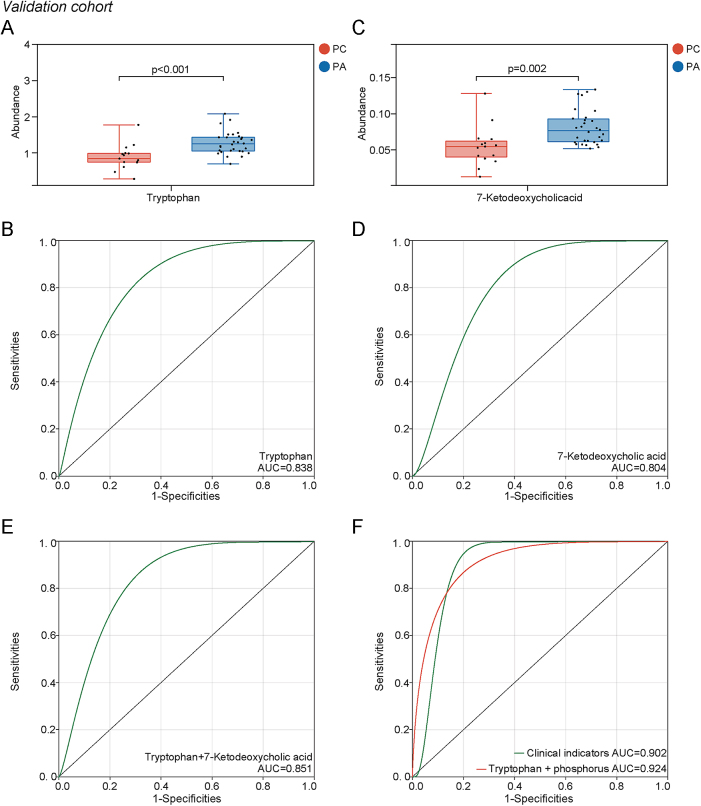
The significance of differentially abundant metabolites in diagnosis of PC and adenomas (PA). (A, B, C, D, E) The ROC curves of confirmed biomarkers in the validation cohort, including tryptophan (AUC = 0.838) and 7-ketodeoxycholic acid (AUC = 0.804), and the combination of tryptophan and 7-ketodeoxycholic acid (AUC = 0.851). (F) Compared with the diagnostic model based solely on clinical indicators (AUC = 0.902), the diagnostic model of tryptophan and serum phosphorus further improves the diagnostic efficacy (AUC = 0.924).

In an attempt to validate the diagnostic efficacy of the potential biomarkers, we collected plasma samples from 15 patients with PC and 30 patients with PA as an external validation cohort before surgery. Among the potential biomarkers mentioned above, two were confirmed as differentially abundant metabolites in the external validation experiment, including 7-ketodeoxycholic acid (*P* = 0.002, AUC = 0.804) and tryptophan (*P* < 0.001, AUC = 0.838), which showed higher accuracy in differential diagnosis in the validation cohort (Table 3) (Fig. 2A, B, C, D). The diagnostic efficacy increased (AUC = 0.851) with the combination of these two endogenous metabolites (Fig. 2E). By multivariable logistic regression, a model including tryptophan (*P* = 0.011) and serum phosphorus (*P* = 0.003) was established to differentiate PC from PA. The AUC value of this diagnostic model was 0.924, which showed better performance than that of the diagnostic model based on clinical indicators (AUC = 0.902) (Fig. 2F). In addition, certain types of metabolites also showed significant differences between the plasma of PCs and PAs in the validation experiment, including phosphatidylinositol (PI) and acylcarnitine (ACar).

**Table 3:** Potential biomarkers detected in the discovery cohort and novel biomarkers verified in the validation cohort.

Potential biomarkers	Discovery cohort		Validation cohort	
	24 PC vs 46 PA		15 PC vs 30 PA	
	*P* values	AUC	*P* values	AUC
8,9-DiHETrE	<0.001	0.762	ND	ND
L-tryptophan	0.020	0.709	<0.001	0.838
LysoPE (0:0/22:4 (7Z,10Z,13Z,16Z))	0.010	0.706	ND	ND
Cortisone	0.011	0.698	ND	ND
FAHFA (19:1/8:0)	0.023	0.694	ND	ND
LPI (20:4)	0.034	0.688	ND	ND
3-phenylpropionic acid	0.035	0.688	ND	ND
9,10-epoxyoctadecanoic acid	0.012	0.678	ND	ND
PI (18:0/20:4)	0.035	0.677	ND	ND
Decanoylcarnitine	0.002	0.673	ND	ND
Thyroxine	0.008	0.672	ND	ND
7-ketodeoxycholic acid	0.014	0.671	0.002	0.804
FAHFA (2:0/22:5)	0.039	0.668	ND	ND
5,6-DHET	0.045	0.667	ND	ND
PI (18:0/18:1)	0.047	0.657	ND	ND
Docosahexaenoic acid	0.045	0.656	ND	ND

ND, not detected; AUC, area under the receiver operating characteristic curve; PC, parathyroid carcinoma; PA, parathyroid adenoma.

### Correlations between differentially abundant metabolites and clinical features

We analysed the relationship between differentially abundant metabolites and clinical features in the discovery cohort. Serum PTH level was correlated with serum calcium level (*R* = 0.637, *P* < 0.001), and serum ALP level (*R* = 0.689, *P* < 0.001). Serum ALP level was correlated with thyroxine (*R* = −0.529, *P* < 0.001) (Fig. 1F). Smoking was associated with the level of isobutyryl-L-carnitine (*P* = 0.028). No significant differences in metabolites were found between males and females. According to the univariate Cox analysis, 8-hydroxy-6,7-dimethoxy-2H-chromen-2-one was identified as a prognostic biomarker for OS of patients with PC (*P* = 0.026). According to the univariate Cox regression, four differentially regulated metabolites were related to DFS, among which 1H-indole-2,3-dione (*P* = 0.001) and (R)-3-hydroxy-tetradecanoic acid (*P* = 0.004) were associated with recurrence according to the multivariate Cox regression.

## Discussion

Although the metabolomics analysis of parathyroid tumour tissue has been investigated previously, this study was the initial exploration of plasma metabolomics in patients with parathyroid neoplasms. In this study, we employed LC–MS/MS analysis to investigate the metabolic signatures of plasma from PC and PA patients. Our findings verified that two endogenous metabolites, 7-ketodeoxycholic acid and tryptophan, showed potential in the differential diagnosis of PC. Moreover, the combined diagnostic efficacy of tryptophan and serum phosphorus outperformed that of the preoperative clinical indicators. In addition, we found that the combination of metabolic biomarkers and postoperative *CDC73* genetic analysis improves the diagnostic efficacy for PC. In the discovery cohort, the metabolic biomarkers showed high diagnostic efficacy (AUC = 0.860), with a sensitivity of 79.2% and a specificity of 82.6%. The diagnostic efficacy increased (AUC = 0.891) with the combination of metabolic biomarkers and *CDC73* abnormality, which presented a sensitivity of 78.3% and a specificity of 95.2%.

In this study, the plasma level of tryptophan was significantly decreased in PC patients. Tryptophan, an essential amino acid, contributes to various pathological and physiological processes. There are three pathways involved in tryptophan metabolism, namely, the kynurenine (Kyn), 5-hydroxytryptamine (HT), and indole pathways (Perez Castro *et al.* 2023). The metabolites in tryptophan metabolism pathways, such as kynurenine and serotonin-derived small compounds, were considered to be biomarkers for diagnosis of both non-neoplasm and neoplasm diseases (Kowalik *et al.* 2022). It is reported that the plasma level of tryptophan combined with melatonin showed remarkable accuracy in the diagnosis of early chronic kidney disease (Fu *et al.* 2024). A random forest model based on tryptophan, kynurenine, and xanthurenic acid could be used to diagnose non-small cell lung cancer with considerable accuracy (Sun *et al.* 2023). Approximately 95% of tryptophan is metabolised through the Kyn pathway, which is highly regulated by a group of key rate-limiting enzymes (Jones *et al.* 2013). The Kyn pathway is highly regulated by key rate-limiting enzymes, such as tryptophan 2,3-dioxygenase (TDO2). TDO2, as one of the key enzymes in the Kyn pathway, was found to be highly expressed in the tumour tissues of PCs (Hu *et al.* 2019). The upregulation of TDO2 was correlated with a worse prognosis in a variety of cancers, including breast, liver, and colon cancers (Chen *et al.* 2016, Li *et al.* 2020, Liu *et al.* 2020). A reduction in tryptophan levels and the activation of kynurenine in tissues inhibits T-cell activities and ultimately leads to immune evasion (Perez Castro *et al.* 2023).

Lower levels of 7-ketodeoxycholic acid in plasma were detected in PC patients. 7-ketodeoxycholic acid is a bile acid derivative and a metabolite generated from the degradation of the primary bile acid, cholic acid, by intestinal bacteria. Accumulating evidence suggested that 7-ketodeoxycholic acid was closely associated with not only digestive, but also non-digestive system diseases. It was reported that higher 7-ketodeoxycholic acid in faecal samples indicated the possibility of colorectal cancer (Khattab *et al.* 2023). The serum level of 7-ketodeoxycholic acid was regulated by gut microbes, which was related to the depression-like symptoms through the gut–brain axis in chronic unpredictable mild stress mice (Ma *et al.* 2022). Besides, 7-ketodeoxycholic acid was also reported to take part in the gut–prostate axis and gut-metabolism-mammary axis (Yu *et al.* 2022, Chen *et al.* 2024). Moreover, a significant difference in the level of 7-ketodeoxycholic acid in faeces was detected between postmenopausal women with osteopenia and postmenopausal women with osteoporosis, which could be used for the distinction of osteopenia and osteoporosis in postmenopausal women (Liang *et al.* 2023). Consequently, more in-depth investigations are imperative to clarify the relationship among gut microbiota, 7-ketodeoxycholic acid, and parathyroid neoplasms.

In PC patients, the significantly enriched pathway related to arachidonic acid metabolism, along with decreased plasma levels of intermediate products in the cytochrome P450 (CYP) monooxygenase pathway in PC patients, including 8,9-DiHETrE and 5,6-DHET, may imply the significance of arachidonic acid metabolism involved in the carcinogenesis of PCs. The CYP pathway is one of three pathways involved in arachidonic acid metabolism. Arachidonic acid metabolism is associated with tumour proliferation and angiogenesis, and ultimately promotes tumour formation and progression. In addition, it is reported that 8,9-DiHETrE and 5,6-DHET were closely related with multiple non-neoplastic diseases. Increased plasma levels of 8,9-DiHETrE indicated an increase in the odds of acute coronary syndrome of 92-fold, and predicted all-cause mortality in patients with severe functional mitral regurgitation (Caligiuri *et al.* 2017, Hofbauer *et al.* 2023). In addition, alterations in plasma 5,6-DHET reflect the incidence of renal dysfunction in uraemic patients and diabetic nephropathy in patients with type 2 diabetes (Hu *et al.* 2018, Peng *et al.* 2021). CYP-dependent epoxygenase and hydroxylase are highly expressed in various cancers (Yarla *et al.* 2016). In a previous microarray study of gene expression in parathyroid neoplasms, significantly increased expression of CYP2D6 was also observed in PCs (Hu *et al.* 2019). Therefore, further exploration is needed on the roles of the key enzymes and intermediate products in the CYP pathway.

Several limitations of this study should be noted. First, although our present cohort was one of the largest PC cohorts reported thus far, the sample size was still limited due to the scarcity of PCs. Second, some plasma specimens were collected from PC patients with recurrent tumours, which may have potential differences from the preoperative specimens and introduce bias. Third, a prospective study of preoperative and postoperative plasma is needed to determine the diagnostic efficacy of metabolite biomarker candidates mentioned above in the future.

## Conclusion

In summary, we performed LC–MS/MS analysis on plasma from patients with parathyroid neoplasms and described the differences in metabolic profiles between PCs and PAs. Tryptophan and 7-ketodeoxycholic acid in plasma could serve as potential biomarkers for the preoperative diagnosis of PC.

## Declaration of interest

The authors declare that there is no conflict of interest that could be perceived as prejudicing the impartiality of the work reported.

## Funding

This work was supported by the Chinese Academy of Medical Scienceshttps://doi.org/10.13039/501100005150 (CAMS) Innovation Fund for Medical Sciences (2021-I2M-1-002), National High-Level Hospital Clinical Research Funding (2022-PUMCH-B-003), and the Peking Union Medical College Hospital Talent Cultivation Program (Category D, UHB12625).

## Author contribution statement

Ya Hu and Ming Cui contributed to the design of the study and critical revision. Jinheng Xiao contributed to the drafting and statistical analysis, and Qingyuan Zheng, Sen Yang, and Tianqi Chen contributed to the data collection and article revision.

## Data availability

The datasets used and/or analysed during the current study are available from the corresponding author upon reasonable request.

## Ethics approval and consent to participate

The study was conducted at the Peking Union Medical College Hospital (PUMCH) and approved by the institutional ethics committee (K2494). Written informed consent was obtained from all patients.
